# Effect of different initiation timing of levothyroxine on pregnancy outcomes and development of offspring in women with gestational subclinical hypothyroidism

**DOI:** 10.12669/pjms.40.10.9724

**Published:** 2024-11

**Authors:** Meirong Fei, Lingnv Shen, Xiuqin Ding, Guoduan Li, Jingyang You

**Affiliations:** 1 Meirong Fei Department of Gynaecology and Obstetrics, Huzhou Nanxun District People’s Hospital, Huzhou, Zhejiang Province 313009, P.R. China; 2 Lingnv Shen Department of Gynaecology and Obstetrics, Huzhou Nanxun District People’s Hospital, Huzhou, Zhejiang Province 313009, P.R. China; 3 Xiuqin Ding Department of Gynaecology and Obstetrics, Huzhou Nanxun District People’s Hospital, Huzhou, Zhejiang Province 313009, P.R. China; 4 Guoduan Li Department of Clinical Laboratory, Huzhou Nanxun District People’s Hospital, Huzhou, Zhejiang Province 313009, P.R. China; 5 Jingyang You Department of Gynaecology and Obstetrics, Huzhou Nanxun District People’s Hospital, Huzhou, Zhejiang Province 313009, P.R. China

**Keywords:** Levothyroxine, Pregnancy outcomes, Subclinical hypothyroidism, Offspring

## Abstract

**Objective:**

To explore the impact of levothyroxine (L-T4) administration at different time points on pregnancy outcomes and offspring development in patients with subclinical hypothyroidism (SCH).

**Methods:**

In this retrospective study, medical records of 107 patients with SCH treated in Huzhou Nanxun District People’s Hospital from February 2021 to March 2023 were retrospectively reviewed. Of them, 55 patients received treatment before eight gestational weeks (Early group), and 52 patients received treatment after eight gestational weeks (Mid group). Levels of thyroid function indicators, occurrence of adverse pregnancy outcomes, occurrence of adverse reactions, and development of offspring before and after the treatment were compared.

**Results:**

After the treatment, the thyroid function indicators in both groups improved compared to before treatment, and the improvement in the Early group was significantly better than that in the Mid group (*P*<0.05). The incidence of adverse pregnancy outcomes in the early group (5.45%) was lower than that in the mid group (19.23%) (*P*<0.05). There was no significant difference in the incidence of adverse reactions between the groups (*P*>0.05). The scores of fine motor, gross motor, language, personal-social behavior, and adaptability in offspring of patients the Early group were higher than those of the mid group (*P*<0.05).

**Conclusions:**

Early administration of L-T4 is safe and can effectively improve thyroid function in SCH patients, reduce adverse pregnancy outcomes, and ensure growth and development of offspring.

## INTRODUCTION

Subclinical hypothyroidism (SCH) is characterized by normal free thyroxine levels that coincide with elevated levels of thyroid-stimulating hormone (TSH).^1^,^2^ SCH occurs in 4%-20% of adults, with higher prevalence among women, older adults, and people with thyroid autoimmunity.^3^ Research has shown that SCH may cause elevated blood lipid and blood pressure levels, thus increasing the risk of adverse maternal and infant outcomes.^1^-^3^ Therefore, timely, safe and effective treatment for SCH is crucial for ensuring favorable maternal and neonatal pregnancy outcomes.

L-thyroxine (*L*-*T4*) is an important alternative treatment drug for SCH patients. It can effectively alleviate clinical symptoms, improve glucose and lipid metabolism and protein synthesis, and plays an important role in maintaining water electrolyte balance.^4^,^5^ According to the American Endocrine Society guidelines, the goal of SCH treatment is to ensure sufficient supply of thyroid hormones during the first rapid brain development period (four to six months of pregnancy) of the fetus.^6^,^7^ Therefore, L-T4 treatment should start before four months of pregnancy, preferably before eight weeks of gestation.^5^-^7^

There have been studies exploring the optimal time of day to administer L-T4,^8^,^9^ but there is currently limited research on the optimal initiation timing for SCH. In addition, most existing studies focus on pregnancy outcomes, while the research on effect of L-T4 timing on the development of offspring is scarce.^10^ Therefore, the purpose of this study was to clarify the clinical value of administering L-T4 treatment at different time point during the pregnancy to provide reference for the optimal intervention time for SCH.

## METHODS

In this retrospective study, medical records of 107 patients with SCH treated in Huzhou Nanxun District People’s Hospital from February 2021 to March 2023 were retrospectively reviewed. Among them, 55 patients received treatment before eight gestational weeks (Early group) and 52 patients received treatment after eight gestational weeks (Mid group).

### Inclusion criteria:

- Met the SCH diagnostic criteria.^11^
- Natural conception, single pregnancy.
- The clinical data is complete.

### Exclusion criteria:

- Patients with autoimmune and metabolic diseases.
- Patients with infectious diseases.
- Patients with thyroid system disorders prior to pregnancy.
- Patients with allergic constitution and a history of allergies to research drugs.
- Patients with malignant tumors.
- Patients with a family history of thyroid diseases.
- Patients with hematological disorders.
- Patients who have taken drugs that affect thyroid function.

### Ethical Approval:

The Ethics Committee of Huzhou Nanxun District People’s Hospital approved this study with the number 2022-083, date: November 15, 2022.

### Treatment:

The initial medication dose of L-T4 (Merck KGaA, Darmstadt, Germany, Approval No. H20100523) was 25 ug/day. The dose was adjusted once every two weeks, with an increase of 25-50 ug/day per week, based on the patient’s thyroid function and clinical symptoms to ensure TSH levels within the target range (first trimester, 0.1–2.5 mIU/L; second trimester, 0.2–3.0. mIU/L; third trimester, 0.3–3.0 mIU/L).

### Observation indicators:

- Thyroid function indicators including thyroid peroxidase antibodies (TPO-Ab), thyroid stimulating hormone (TSH), free thyroxine (FT4), free triiodothyronine (FT3), thyroid hormone (T4), and triiodothyronine (T3) were measured in the serum of 4 ml of fasting venous blood by fluorescence immunoassay using appropriate kits (Boster Biological Technology Co. Ltd; China).
- Adverse pregnancy outcomes such as low birth weight infants, fetal distress, placental abnormalities, premature birth, and miscarriage.
- The occurrence of adverse reactions such as sweating, insomnia, dizziness, nausea, vomiting, and palpitations.
- Development status of offspring: newborns at the age of three months were evaluated based on the Developmental Scale for Children Aged 0-6 Years (WS/T 580-2017).^12^ Assessment included gross motor, fine motor, language, personal-social behavior, and adaptability. Developmental quotient scores were combined to obtain a comprehensive developmental quotient (DQ). DQ=developmental age/actual age × 100.^12^ Based on the score, DQ range is divided into excellent (≥ 130 points), good (115-129 points), moderate (85-114 points), low (70-84 points), and developmental delay (≤ 69 points).

### Statistical analysis:

All data analysis was conducted using SPSS 22.0 software (IBM Corp, Armonk, NY, USA). The normality of the data was evaluated by Shapiro Wilk test. The data of normal distribution were represented by mean ± standard deviation, independent sample *t*-test was used for inter group comparison, and paired *t*-test was used for intra group comparison before and after the treatment. The data with non-normal distribution was represented by median and interquartile intervals. Mann Whitney *U* test was used for inter group comparison, and Wilcoxon signed rank test was used for intra group comparison before and after the treatment. The counting data was represented by the number of cases using chi square test. A *p*-value less than 0.05 was considered statistically significant. All reported *p*-values were bilateral.

## RESULTS

The age of the patients in the early group was 21-39 years, with a median age of 28 (25-32) years. In terms of the previous delivery status, 36 were primiparous and 19 were multiparous women. The body mass index (BMI) was 19.7~29.5 kg/m^2^, with an average BMI of 24.99±2.44 kg/m^2^. The age of the patients in the mid group was 23-38 years, with a median of 29.5 (26-32) years. A total of 34 patients were primiparous and 18 were multiparous. The BMI was 19.4~29.9 kg/m^2^, with an average of 24.57±2.67 kg/m^2^. There was no significant difference in general data such as age, previous delivery status, and BMI between the two groups (*P*>0.05).

**Fig.1:**
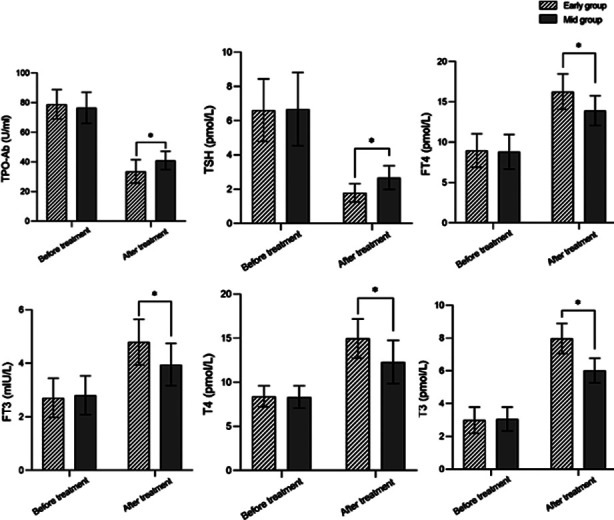
Fig.1: Comparison of thyroid function indicators between two groups. Note: Compared with the Mid group, *P<0.05. TPO-Ab = thyroid stimulating hormone; FT4 = free thyroxine; FT3 = free triiodothyronine; t4 = thyroid hormone; T3 = triiodothyronine.

Before the treatment, there was no significant difference in the levels of TPO-Ab, TSH, FT4, FT3, T4, and T3 between the two groups (*P*>0.05). After the treatment, the levels of TPO-Ab and TSH in both groups decreased compared to before treatment and were lower in the early group compared to the Mid group. Levels of FT4, FT3, T4, and T3 increased compared to before treatment, and were significantly higher in the Early group (*P*<0.05) (Fig.1).

The incidence of adverse pregnancy outcomes in the Early group (5.45%) was lower than that in the mid group (19.23%) (*P*<0.05) (Table-I). There was no significant difference in the incidence of adverse reactions between the Early (5.45%) and the mid group (7.69%) (*P*>0.05) (Table-II). The scores of fine motor, gross motor, language, personal-social behavior, and adaptability in the Early group were higher than those in the Mid group (*P*<0.05) (Table-III).

**Table-I:** Comparison of adverse pregnancy outcomes between two groups [n (%)].

Group	n	Low birth weight infants	Fetal distress	Placental abnormality	Premature birth	Abortion	Total occurrence rate
Early group	55	1 (1.82)	1 (1.82)	0 (0.00)	1 (1.82)	0 (0.00)	3 (5.45)
Mid group	52	3 (5.77)	2 (3.85)	1 (1.92)	3 (5.77)	1 (1.92)	10 (19.23)
*χ^2^*							4.753
*P*							0.029

**Table-II:** Comparison of adverse reactions between two groups [n (%)].

Group	n	Excessive sweating and insomnia	Dizzy	Nausea	Vomit	Palpitate	Total occurrence rate
Early group	55	1 (1.82)	0 (0.00)	1 (1.82)	1 (1.82)	0 (0.00)	3 (5.45)
Mid group	52	0 (0.00)	1 (1.92)	2 (3.85)	0 (0.00)	1 (1.92)	4 (7.69)
*χ^2^*							0.219
*P*							0.640

**Table-III:** Comparison of Development Status of Two Groups of Offspring (Score).

Group	n	Fine motor	Gross motor	language	Personal-Social behavior	Adaptability
Early group	55	108 (102-115)	109 (103-116)	106 (102-113)	103 (99-110)	101 (97-108)
Mid group	52	102 (95.5-106)	101 (94.5-105)	98 (91.5-102)	97 (91-101)	95 (89-99)
*t*		-3.522	-4.429	-4.672	-3.993	-4.149
*P*		<0.001	<0.001	<0.001	<0.001	<0.001

## DISCUSSION

L-T4 is an artificially synthesized sodium salt of tetraiodothyronine that acts similarly to thyroid hormone, secreted by the thyroid.^13^,^14^ Our study found that compared to patients who received treatment after eight gestational weeks (the Mid group), patients in the Early group who were treated before eight gestational weeks showed better improvement in thyroid function indicators, indicating that early adoption of L-T4 treatment can more effectively regulate thyroid hormone levels. The incidence of adverse pregnancy outcomes in patients who received early L-T4 therapy was lower than in patients who were treated with L-T4 after eight weeks of gestation, indicating the importance of screening for SCH, timely diagnosis of the disease, and corresponding treatment. Early L-T4 therapy can improve and restore thyroid function, which ensures normal gestation period, thereby reducing the occurrence of adverse pregnancy outcomes such as low birth weight infants and premature delivery.^15^,^16^ Therefore, the results of our study indicate that L-T4 is safe, and has high therapeutic value in SCH. Our study demonstrated that early administration of L-T4 can effectively improve patient thyroid function, and is associated with positive pregnancy outcomes and maternal and infant health, which is generally consistent with the results by Zhao et al^17^, and Ju et al.^18^

Leng et al.^19^ explored the application value of L-T4 in patients with SCH, and confirmed that it can effectively reduce the rate of neonatal asphyxia and premature birth, and improve the natural delivery rate. It is suggested that L-T4 can improve patient thyroid function, reduce the risk of adverse pregnancy outcomes, and increase the natural delivery rate. The research results of Sun et al.^20^ showed that the levels of thyroid hormones (FT3, FT4, and TSH) in the early intervention group were higher than those in the middle intervention group after the treatment, and were associated with improved maternal and infant health and pregnancy outcomes, which is consistent with the results of this study. Runkle et al.^21^ confirmed that early standardized treatment for SCH patients can effectively reduce the occurrence of related complications and improve maternal outcomes. Ding H et al.^22^ explored the differences in the effectiveness of treatment for SCH patients at different time points and showed that the incidence of pregnancy diabetes and hypertension (20.9%, 18.6%), neonatal asphyxia (2.3%), and premature delivery (7.0%) in the early treatment group were lower than those in the late treatment group.

Moreover, the DQ scores (93.3±3.3 and 94.6±2.8) of the early group’s offspring at 16th and 28th weeks, respectively, were significantly higher than those in the late treatment group. Our study also found that the offspring of patients in the Early group had higher scores for fine motor, gross motor, language, personal-social behavior, and adaptability compared to the Mid group. Together with previous reports, our results further confirm that early onset of L-T4 treatment not only improves maternal and infant health outcomes, but also has a positive effect on ensuring the healthy growth and development of offspring.

### Limitations:

Firstly, it is a single center retrospective study. Secondly, neither group was randomly assigned. Therefore, baseline information may be imbalanced and biased, which is also one of the shortcomings of a retrospective study. Thirdly, although we tried to reduce confounding factors, there may be unmeasurable variables and residual confounding factors in the results. Finally, the impact of the two treatments on the long-term functional recovery of the mother and infant was not analyzed. Further higher quality studies are needed to verify our conclusions.

## CONCLUSION

Early administration of L-T4 can effectively improve thyroid function in SCH patients, reduce adverse pregnancy outcomes, ensure neonatal growth and development, and is safe.

### Authors’ contributions:

**MF:** Conceived and designed the study.

**MF**, **LS**, **XD**, **GL** and **JY:** Collected the data and performed the analysis.

**MF:** Was involved in the writing of the manuscript and is responsible for the integrity of the study.

All authors have read and approved the final manuscript.
